# Suspected Malignant Hyperthermia and the Application of a Multidisciplinary Response

**DOI:** 10.3390/healthcare8030328

**Published:** 2020-09-09

**Authors:** Laura Ebbitt, Eric Johnson, Brooke Herndon, Kristina Karrick, Aric Johnson

**Affiliations:** 1 Department of Pharmacy, University of Kentucky Medical Center, Lexington, KY 40536, USA; Laura.Means@uky.edu (L.E.); Bherndon01@uky.edu (B.H.); Kristina.Huey@uky.edu (K.K.); 2 Department of Anesthesiology, University of Kentucky Medical Center, Lexington, KY 40536, USA; Aric.Johnson@uky.edu

**Keywords:** malignant hyperthermia, collaborative practice, perioperative care

## Abstract

Purpose: Malignant hyperthermia (MH) is a critical and potentially life-threatening emergency associated with inhaled anesthetic and depolarizing neuromuscular blocker administration. This is a single center’s response to MH. Summary: When signs of MH are observed, a page for “anesthesia STAT-MH crisis” is called, triggering a multidisciplinary response, including the deployment of a Malignant Hyperthermia Cart. The MH cart and the delegation of duties allows nurses, physicians and pharmacists to quickly understand their role in the stabilization, transition and recovery of a suspected MH patient. Conclusion: This case highlights the importance of multi-disciplinary involvement in these rare, but potentially fatal, cases.

## 1. Introduction

Malignant hyperthermia (MH) is a critical and potentially life-threatening emergency associated with the administration of volatile anesthetics and depolarizing neuromuscular blockers that may occur intraoperatively, as well as during the postoperative period [1]. It is treated with dantrolene, a ryanodine receptor antagonist. Both the Malignant Hyperthermia Association of the United States (MHAUS) and American Society of Anesthesiologists (ASA) emphasize a preemptive approach to treatment, including MH supply, a medication cart and departmental training [2,3]. Furthermore, delays between the onset of MH and a coordinated response involving the administration of dantrolene have been associated with increased rates of complications [1]. Therefore, a rapid and efficient response to those with suspected MH may limit the morbidity associated with the condition. We present a case of suspected MH and illustrate the application of a multidisciplinary response in accordance with a well-rehearsed institutional protocol.

### Pathophysiology

Malignant hyperthermia is an autosomal-dominant, pharmacogenetic disorder that manifests as a hypermetabolic crisis following exposure to a triggering agent. Known triggering agents include all volatile anesthetics (isoflurane, sevoflurane and desflurane), depolarizing neuromuscular blocking agents (succinylcholine) and human stressors such as vigorous exercise and heat [4]. The most common genetic mutation found to cause MH involves changes to the type 1 ryanodine receptor (RYR1), which encodes for the ryanodine receptor found on skeletal muscle [5]. The RYR1 is located on the sarcoplasmic reticulum of myocytes and is essential for regulating muscle excitation–contraction coupling. In the setting of genetic mutation and a triggering agent, rapid and uncontrolled increases in myoplasmic calcium occur, although this may not occur in the patients’ initial surgeries. This is significant, as both metabolism and contraction in skeletal muscle are regulated by the concentration of intracellular calcium [6]. Manifestations of the dysregulation are indicative of a hypermetabolic state. These derangements may occur as early or late signs. Early signs may include sudden elevated end-tidal carbon dioxide, tachycardia, acidosis and muscle rigidity. Late signs may include hyperthermia and hyperkalemia [4]. If untreated, these symptoms may progress to rhabdomyolysis, myoglobinuria and acute renal failure. Life-threatening complications include disseminated intravascular coagulopathy (DIC), congestive heart failure, bowel ischemia and compartment syndrome [4]. The prompt diagnosis and treatment of MH is key to preventing the progression of symptoms and avoiding significant morbidity or death.

## 2. Institutional Approach/Protocol

**Figure 1:**
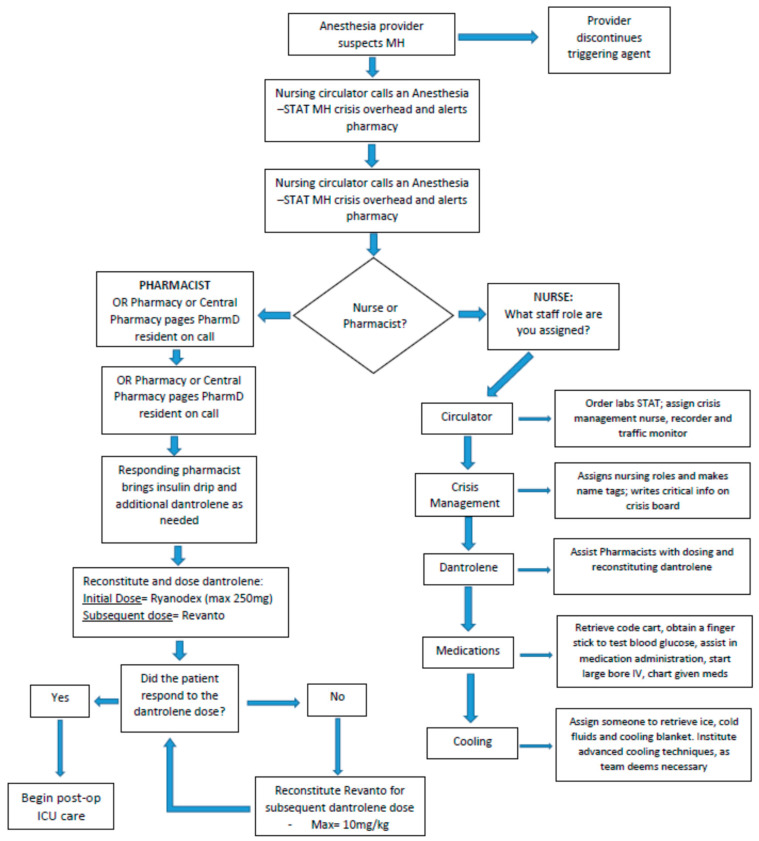
Sequence of events involved in Malignant Hyperthermia Response.

Prior to the administration of any anesthetic, all patients should be screened for MH through a complete medical and family history analysis. This may not be possible in emergency situations. The initial signs of MH may occur at any time following the administration of a triggering agent, including immediately following the induction of general anesthesia or at any point during the maintenance phase for the anesthetic. As previously mentioned, the earliest clinical signs include an increase in the end-tidal carbon dioxide and tachycardia. As these findings are much more frequently a result of inadequate anesthesia and hypoventilation, respectively, the anesthesiologist must maintain a high level of suspicion for MH. If the anesthesiologist feels that MH is probable, or if there is no alternative diagnosis to explain the patient’s clinical findings, they should immediately discontinue any triggering agents, notify the surgeon, hyperventilate with 100% inspired oxygen, increase fresh gas flow to >10 L/min, and trigger our multidisciplinary response. If available, charcoal filters should also be placed on the inspiratory and expiratory limbs of the anesthesia circuit. As MH is a potentially lethal disorder, a well-coordinated multidisciplinary approach is valuable in ensuring a timely and organized response. Figure 1 demonstrates the sequence of events initiated at our institution when MH is suspected.

An “anesthesia stat-MH crisis” is called out over the intercom to alert operating room (OR) staff including anesthesiologists, nurses and pharmacists to respond and assist in treating the patient. The anesthesiologist will serve as the primary leader for the resuscitation response and ensure that all aspects of patient care are accounted for. The primary OR nurse will retrieve the MH cart (contents shown in Table 1) from the adjoining storage area and bring it into the OR. Color-coded cards corresponding to tasks or roles are assigned to responding personnel. These roles include a registered nurse (RN) circulator, cooling nurse, medication nurse, dantrolene nurse/pharmacist and crisis management nurse. Attached to each card is a bag of supplies specific to the individual’s role. The RN circulator may assign additional MH Team roles as needed. The cooling nurse procures ice and is prepared to implement advanced cooling as indicated. Cooling techniques at our institution include ice bags at the groin, axilla and neck; cooling blankets; and cold saline, as indicated. The medication nurse starts a large bore IV and works with the pharmacist to calculate the appropriate dantrolene dose. The pharmacist double-checks all drug dosing and assists with medication documentation, as well as ensuring the order of dantrolene products are utilized in the correct order to maximize efficiency and cost-effectiveness. Additionally, the pharmacists help to procure regular insulin and dextrose if needed for the treatment of hyperkalemia. Without all of these providers assessing and participating in the care of the patient, these cases would be extremely laborious. Having a multidisciplinary team attend to an MH crisis allows for the rapid control of a patient’s symptoms and to potentially stabilize them quickly.

Two types of dantrolene are contained in our MH cart, one vial of Ryanodex and nine vials of Revonto, in addition to the 10 vials of nonbacteriostatic sterile water (nine 100 mL vials and one 20 mL vial). The Ryanodex is used for the first dose, and the nine vials of Revonto are provided for any necessary subsequent dosing. Ryanodex is a lyophilized powder form of dantrolene containing 250 mg per vial, which costs around USD 2500.00. Revonto is also a lyophilized powder but in contrast only contains 20 mg of dantrolene per vial, costing around USD 60.00 a vial. To reconstitute Ryanodex, only 5 mL of sterile water is required. When reconstituting Revonto, 60 mL is needed per vial. Ryanodex should be used for the first dose because of its ease of use and need to reconstitute fewer vials. For an average 80 kg patient, 10 vials of Revonto and 600 mL of sterile water would be required to reconstitute an initial dose. Other components of the MH cart are listed below in Table 1 and follow MHAUS recommendations [7].

Once the patient’s MH symptoms and the patient are clinically stabilized, post-operative critical care and intensive care unit (ICU) admission are initiated. Patient allergies are updated to include likely triggering agents as a placeholder for future operations and hospital visits as a safety measure. When appropriate, patient and family are counseled on the importance of notifying anesthesia providers about MH history and avoiding triggering agents.

## 3. Case

With the consent of the patient, we present a case of a 22-year-old male admitted with an open right intercondylar fracture of the distal humerus after getting his arm caught in a steel press. In the emergency department, the patient received intravenous cefazolin, morphine, hydromorphone and a Tetanus/Diptheria/Pertussis (Tdap) vaccine. He was taken to the OR on the same day for an irrigation and debridement, as well as closed reduction of the open distal humerus fracture. General anesthesia was induced with lidocaine, fentanyl and propofol. Rocuronium was used for neuromuscular blockade. During the procedure, he was maintained on sevoflurane. No complications were noted during or after initial surgery. The following day, the patient was scheduled for a definitive internal fixation of his distal humerus fracture. General anesthesia was again induced with lidocaine, fentanyl and proprofol. Succinylcholine was administered to facilitate endotracheal intubation. The patient was maintained on isoflurane, and intermittent dosing of rocuronium was used to facilitate neuromuscular blockade. Approximately 30 min into the procedure, during the placement of an additional intravenous line, unexpected resistance was noted. Upon closer examination, his extremities were found to be rigid. A quick assessment of his vitals showed that he was tachycardic, with a heart rate of 160 bpm; hypercapnic, with an end-tidal CO_2_ of 62 mmHg; and hypertensive, with systolic blood pressures >160 mmHg (baseline blood pressure was 130/82 mmHg at preoperative evaluation). A temperature-sensing catheter was placed in the bladder, and the patient was found to be normothermic at 37.4 °C. Despite the normothermia, malignant hyperthermia was suspected. The isoflurane was discontinued, and charcoal filters were placed in the circuit. Nitrous oxide was used to maintain general anesthesia, and a malignant hyperthermia response was initiated and allowed for additional responders to arrive at the patient’s bedside within minutes.

The patient quickly received an initial bolus of 187.5 mg of Ryanodex (2.5 mg/kg). Additionally, 20 mg of IV push esmolol was administered to treat his tachycardia but with a negligible response. Over the next 35 min, the patient received 80 mg of Revonto via intermittent 20 mg doses. These doses were administered to treat persistent and intermittent symptoms of MH.

The non-pharmacological measures taken include ice packs applied to the axilla and the placement of cooling blankets. The patient responded to the dantrolene with marked reductions in heart rate, muscle rigidity and end-tidal carbon dioxide (EtCO_2_). While not elevated, the patient’s temperature remained normothermic. The surgical procedure was aborted, and the patient was transferred to the ICU for close monitoring, with care being assumed by the ICU intensivists.

**Figure 2:**
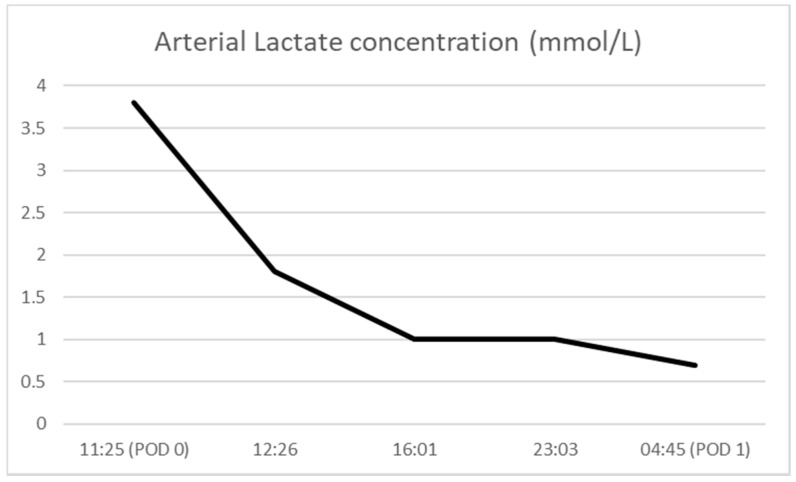
Arterial lactate concentration (POD = post-operative day).

**Figure 3:**
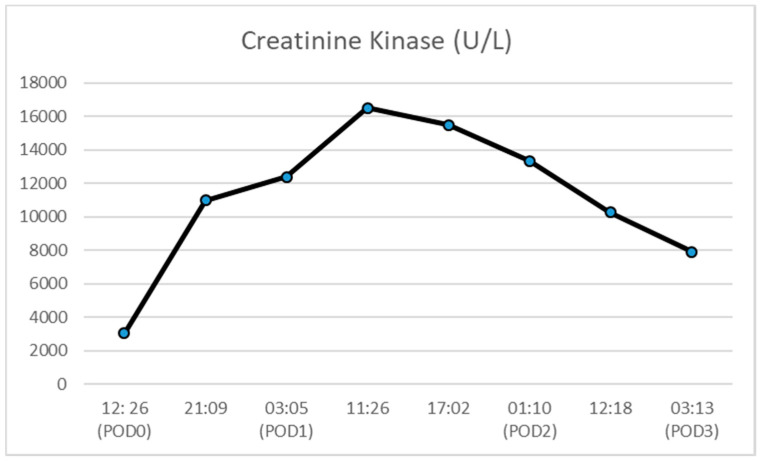
Creatinine kinase concentration (POD = post-operative day).

In the ICU, the patient continued to receive Revonto 80 mg (~1 mg/kg) IV Q 6 h for 24 h. During this time period, the patient’s lactate fell from 3.2 mmol/L at its peak to 0.6 mmol/L (Figure 2). His creatinine kinase (CK) peaked at 16,505 units/L and decreased to 7887 units/L prior to discharge (Figure 3). The patient’s serum creatinine (SCr) was also elevated at 1.44 mg/dL and trended back down to his assumed baseline.

In light of the etiology and triggering factor in this case, one may incorrectly assume it was precipitated by succinylcholine alone, since the patient previously received sevoflurane without incident. However, the literature suggests that different inhaled anesthetics may trigger MH at different rates, and his initial sevoflurane exposure was not sufficient [8]. Furthermore, studies have shown that a triggering inhalation agent plus the use of succinylcholine may cause a more marked response than a single agent [9].

After the patient was stabilized, the case was discussed with the mother, who had also experienced MH in the past; however, she was not aware that this was a hereditary disease. The patient’s family was educated regarding the risks of MH and the potential for genetic predisposition within the family. An allergy was also added to the patient’s chart for future potential cases. The patient was extubated that evening. Four days into the patient’s admission, he received an open reduction internal fixation (ORIF) of his distal humerus. Total IV anesthesia (TIVA) was used with continuous infusion of propofol and intermittent dosing of fentanyl, dexmedetomidine and rocuronium throughout the case. Aside from the CK, lactate and SCr, the patient’s lab results all remained normal, and the patient progressed to his baseline function. The patient was discharged home on post-operative day 3 from the index surgery, with follow up after 2 weeks with the orthopedic service. Through the utilization of the institution’s protocol, all providers were aware of their roles within the team and were able to quickly perform their assigned duties. This allowed delays to be reduced for the rapid control of the patient’s MH. Without the swift initiation of an MH protocol, it is possible that patients could experience a lethal outcome.

## 4. Conclusions

MH is a rare but serious metabolic complication associated with the use of volatile anesthetics and depolarizing neuromuscular blocking agents. In the case of a delayed response or missed diagnosis, significant morbidity and mortality may occur. Institutions should develop, implement and train staff on how to recognize and treat this acute disorder. We present the case of a patient with an unknown family history of malignant hyperthermia. Despite proper pre-operative assessment, the family history was missed, and the patient experienced MH symptoms after receiving a triggering agent during his second surgery. Due to an extensive, multidisciplinary perioperative MH protocol, this patient was successfully treated and avoided serious complications. Providers were able to treat the patient quickly and efficiently, in great part due to the presence and utilization of the MH cart. The dosing cards and instructions readily available on the cart allowed the correct dose of Ryanodex to be verified and drawn up into a syringe by the providers while subsequent doses of Revonto were also being prepared. This case also highlights the need to ask specific questions in the pre-operative setting regarding both the patient’s and the patient’s family’s prior history of surgeries and any events that may have occurred. We recommend that other institutions develop a similar cart, as a mechanism for providers to be able to respond to these events.

## Figures and Tables

**Table 1:** Contents of Malignant Hyperthermia Cart.

Medications	Anesthesia Supply	Nursing Supplies
10% calcium chloride (1000 mg/10 mL) syringe (3)	Central line kit	Salem sump
Dextrose, 5%, syringe (1)	Arterial line kit	Rapid Infusion Catheter (RIC)
Sodium bicarbonate, 8.4% (2)	Charcoal gas machine filter	Temperature-sensing Foley
Sterile water, 50 mL (9)	Guidewires	Pressure bag
Revonto (dantrolene), 20 mg/60 mL (9)		
Ryanodex (dantrolene), 250 mg/5 mL (1)		
